# Effectiveness of beta-blockers depending on the genotype of congenital long-QT syndrome: A meta-analysis

**DOI:** 10.1371/journal.pone.0185680

**Published:** 2017-10-23

**Authors:** Jinhee Ahn, Hyun Jung Kim, Jong-Il Choi, Kwang No Lee, Jaemin Shim, Hyeong Sik Ahn, Young-Hoon Kim

**Affiliations:** 1 Division of Cardiology, Department of Internal Medicine, Korea University College of Medicine and Korea University Medical Center, Seoul, Republic of Korea; 2 Division of Cardiology, Department of Internal Medicine, Pusan National University Hospital, Busan, Republic of Korea; 3 Department of Preventive Medicine, Korea University College of Medicine, Seoul, Republic of Korea; University of Tampere, FINLAND

## Abstract

**Background:**

Beta-blockers are first-line therapy in patients with congenital long-QT syndrome (LQTS).

**Objective:**

This study sought to determine the differences in effectiveness of beta-blockers on risk reduction according to LQTS genotype.

**Methods:**

We searched MEDLINE, EMBASE, and CENTRAL databases to investigate the use of beta-blockers (atenolol, nadolol, propranolol, and metoprolol) in patients with LQTS. Hazard ratio (HR) and relative risk (RR) were extracted or calculated from studies reporting cardiac events (syncope, aborted cardiac arrest (ACA), or sudden cardiac death (SCD)).

**Results:**

Among 2,113 articles searched, 10 studies (7 registry-based cohort studies (Cohort) and 3 interrupted time series studies (ITS)) involving 9,727 patients were included. In a meta-analysis using a random-effect model, the use of beta-blocker was associated with significant risk reduction of all cardiac events (HR 0.49, p<0.001 in Cohort; RR 0.39, p<0.001 in ITS) and serious cardiac events (ACA or SCD) (HR 0.47, p<0.001 in Cohort). In both LQT1 and LQT2, the risk was reduced with beta-blocker therapy in Cohort (HR 0.59 in LQT1; HR 0.39 in LQT2) as well as ITS (RR 0.29 in LQT1; RR 0.48 in LQT2). Among the beta-blockers, nadolol showed a significant risk reduction in both LQT1 and LQT2 (HR 0.47 and 0.27, respectively), whereas atenolol and propranolol decreased the risk only in LQT1 (HR 0.36 and 0.46, respectively). Metoprolol showed no significant reduction in either genotype. In LQT3, beta-blocker therapy was not as effective as LQT1 or LQT2; however, it was inconclusive due to data insufficiency.

**Conclusion:**

This meta-analysis showed that beta-blockers were effective in reducing risk of cardiac events in patients with LQTS. Among them, nadolol was effective in LQT1 and LQT2, whereas other drugs showed different effectiveness depending on LQT genotype.

## Introduction

Congenital long-QT syndrome (LQTS) is one of the most common inherited arrhythmia syndromes and was described for the first time in 1957 [1]. LQTS is characterized by abnormal QT-interval prolongation on surface electrocardiogram (ECG) and increases risk of development of polymorphic ventricular tachycardia, predisposing the patient to syncope, aborted cardiac arrest (ACA), and sudden cardiac death (SCD) [2]. Three major genes, LQT1, LQT2, and LQT3, constitute more than 80~90% of the 15 genotypes reported in LQTS [3].

The use of beta-blocker is a mainstay of treatment for LQTS [4]. The mechanism of beta-blockers to prevent cardiac events is generally considered to be related to an anti-adrenergic effect, and their efficacy has been shown in previous studies [5–7]. However, previous observational data demonstrated differing efficacy among the drugs. These differences could be attributed to LQTS subtype and the specific beta-blocker [8–10]. The concept of a genotype-phenotype mechanism has been proposed as cardiac events in each subtype of LQTS are associated with gene-specific triggers [11]. That is, beta-blocker use is considered the most effective in patients harboring LQT1, which is closely related to increased sympathetic tone, whereas the drugs are least effective, even harmful, in patients with LQT3, in which rate-dependent fatal ventricular arrhythmia occurs. On the contrary, protective effects of beta-blockers have been demonstrated in females with LQT3 [12–14]. In addition, the various pharmacological and pharmacokinetic properties of different beta-blockers can also affect the efficacy even in the same LQT subtype [15].

However, the effectiveness of different β-blockers for reducing cardiac events according to LQTS genotype remains little known. Most publications regarding beta-blocker therapy in patients with LQTS have been case reports or registry-based studies, rendering the proper choice of beta-blocker difficult [4]. Thus, the current guideline does not recommend one beta-blocker over the others. These reports raise questions about whether or not various β-blockers have different efficacies on reduction of cardiac events depending on genotypes. Here, we systematically reviewed the available data from observational studies to determine the effectiveness of different beta-blockers on risk reduction depending on genotype in patients with LQTS using meta-analysis.

## Materials and methods

### Data sources and searches

We followed the guidelines for meta-analysis according to the Preferred Reporting Items for Systematic Reviews and Meta-Analyses (PRISMA) statement (S1 Checklist) [16]. All articles were searched from computerized databases including MEDLINE, EMBASE, and the Cochrane Library (CENTRAL) as well as manually searching for sources of potentially relevant information. The search was restricted to clinical articles pertaining humans and studies written in English between 1957 and Dec 2016. Article review was performed by two independent investigators (J.A. and J.-I.C.); disagreement was adjudicated by a third. Search strategy details are shown in S1 Table.

### Definition and study eligibility

**Fig 1:**
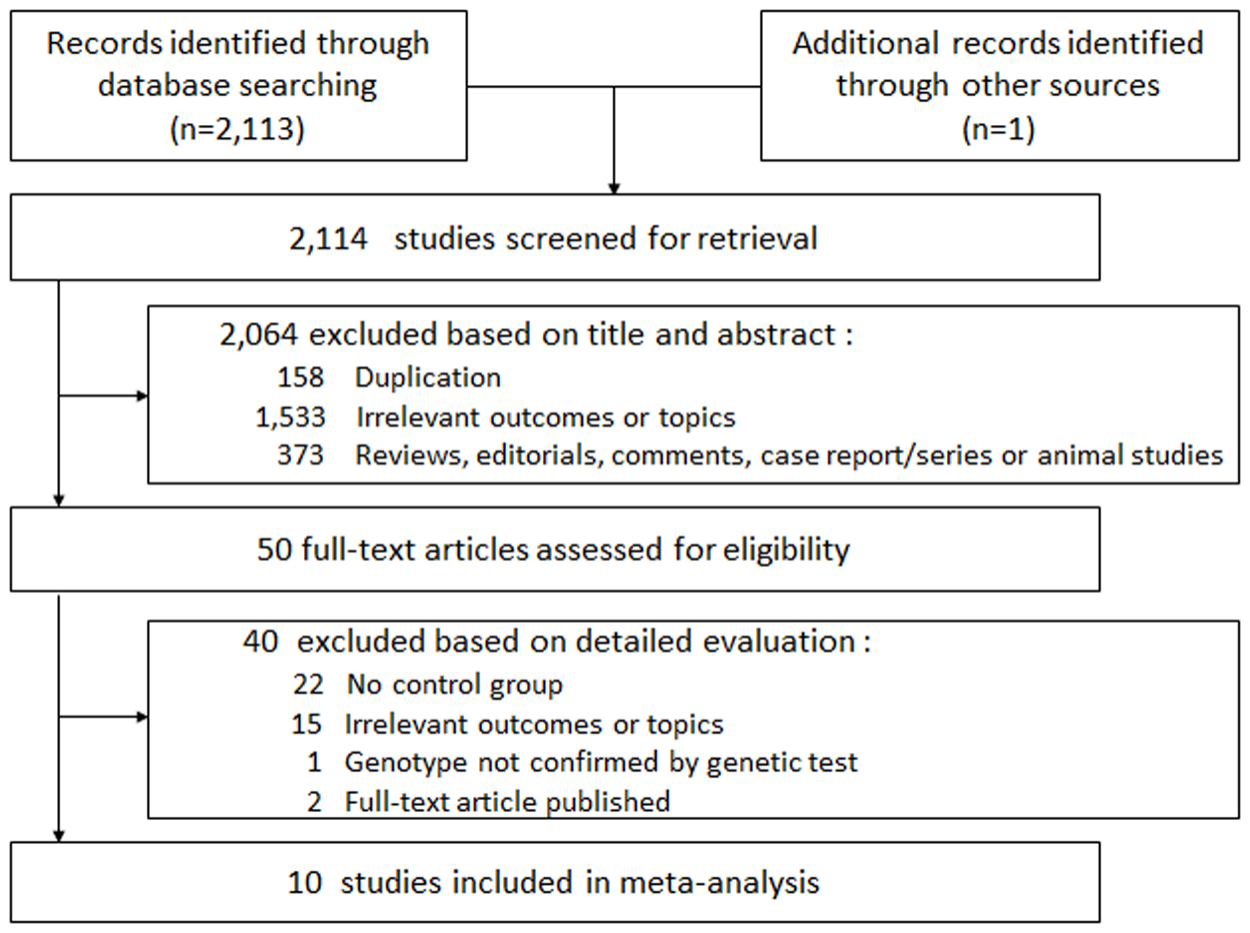
Flow diagram of the literature search and study selection.

Definition of primary outcome in this study was the effect of beta-blocker on cardiac events in congenital LQTS. Cardiac event was defined as syncope, ACA, or SCD. The inclusion criteria were as follows: (1) Included participants diagnosed with congenital LQTS based on genetic test, (2) Investigated the effect of beta-blockers on cardiac events compared with a group not treated with beta-blocker, and (3) Suggested the effects of beta-blockers as relative risk (RR), odds ratio (OR), or hazard ratio (HR). Two investigators screened the titles and abstracts and excluded all papers not fitting the criteria above. Each remaining study was reviewed in full text to select final eligible studies (Fig 1).

### Data extraction

From included studies, extracted data included authors, study type, year of publication, title, country of origin, number of participants, age, gender, enrollment period in registry-based studies, distribution of LQT genotypes, number of participants based on beta-blocker use, and effects of beta-blockers (number of event occurrences, RR, OR, or HR). We tried to contact the corresponding authors to obtain further information via e-mail.

### Quality assessment

Study quality was assessed by the same two independent investigators using a Newcastle-Ottawa Quality Assessment Scale for registry-based cohort studies (Cohort) [17, 18] and 7 standard criteria suggested by the Effective Practice and Organization of Care group for interrupted time series studies (ITS) [19, 20]. Any disagreement was adjudicated by the third reviewer.

### Statistical analysis

The meta-analysis was conducted in line with recommendations from the Cochrane Collaboration and the Quality of Reporting of Meta-analyses guidelines and was performed according to the PRISMA statement [16]. We used the Mantel-Haenszel random-effects model to investigate the effects of beta-blockers on cardiac events in congenital LQTS throughout the trials [21, 22]. HRs or RRs with 95% confidence intervals (CIs) were calculated or recalculated for each study and pooled in the random effects models. The analysis was conducted in Cohort and ITS studies separately since the study design was quite different. Heterogeneity among studies was formally assessed using Q and *I*^*2*^ statistics. Two-sided p value < 0.05 was considered statistically significant. Statistical analyses were performed using the RevMan 5.3 (Cochrane Collaboration, Oxford, United Kingdom) and SPSS Statistics 19.0 software (SPSS Inc., Armonk, NY, USA). Specific approval for meta-analysis is not required by either the Institutional Review Boards or Korea University Medical Center. Funnel plotting for publication bias was not required because the number of analyzed studies in each analysis was less than 10.

## Results

### Search results

Fig 1 depicts the process of identifying eligible articles. A total of 2,114 studies were retrieved for screening analysis. After review based on titles and abstracts, 2,064 studies were excluded since they were duplicated or irrelevant to our purpose. The remaining 50 studies were reviewed for eligibility in full text. After detailed evaluation, 40 studies with neither a control group nor relevant measurements of the outcome were excluded. Finally, 10 studies were included in the meta-analysis [5, 7–9, 14, 23–27].

### Study characteristics

The characteristics of the 10 included studies are summarized in Table 1. Of these, 7 were Cohort and 3 were ITS. The total number of participants in this meta-analysis was 9,727, and more patients were female. All participants in the studies were diagnosed with LQTS confirmed by genetic testing. 4 studies included a single LQT genotype, whereas the remaining studies contained two or more genotypes. In all studies, beta-blockers were prescribed in the treated group; the drugs were specified in 6 studies and were typically atenolol, nadolol, metoprolol, or propranolol. Quality assessment is depicted in S1 Fig. Overall, 78.3% of the bias items were rated as low risk.

**Table 1:** Characteristics of studies included in the analysis of the effects of beta-blockers on cardiac events.

Study (Ref. no)	Study design	No. of participants		Mean age, yrs	Male, %	Follow-up duration, yrs	Included LQTS genotype(s)	Prescribed ßB(s)	Definition of cardiac events
		ßB use	No ßB						
Goldenberg et al. (2008) [7]	Cohort	643	2472	7.5	37	11.4	LQT1, LQT2, LQT3	atenolol, nadolol, metoprolol, propranolol	ACA, SCD
Shimizu et al. (2009) [23, 28]	Cohort	858 (NS)		27.2	40.2	ND	LQT2	NS	Syncope, ACA, SCD
Goldenberg et al. (2010) [24, 29]	Cohort	971	415	16	41.5	31	LQT1	atenolol, nadolol, metoprolol, propranolol	Syncope, ACA, SCD, ICD shock
Goldenberg et al. (2012) [25]	Cohort	333	388	ND	34.6	30	LQT1, LQT2	NS	Syncope, ACA, SCD
Abu-zaitone et al. (2014) [9]	Cohort	1319	201	14.9	38.3	ND	LQT1, LQT2	atenolol, nadolol, metoprolol, propranolol	Syncope, ACA, SCD
Koponen et al. (2015) [27]	Cohort	244	72	5.8	46.8	12	LQT1, LQT2	atenolol, bisoprolol, metoprolol, propranolol	Syncope, ACA, SCD
Wilde et al. (2016) [14]	Cohort	111	280	28	45	7.25	LQT3	NS	Syncope, ACA, SCD
Moss et al. (2000) [5]	ITS	869		15.7	41	5	LQT1, LQT2, LQT3	atenolol, nadolol, metoprolol, propranolol	Syncope, ACA, SCD
Priori et al. (2004) [8]	ITS	335		26	37.9	4.7	LQT1, LQT2, LQT3	NS	Syncope, ACA, SCD, VT/TdP
Vincent et al. (2009) [26]	ITS	216		26	36	10	LQT1	atenolol, nadolol, metoprolol, propranolol, others*	Syncope, ACA, SCD

ßB = beta-blocker; LQTS = long-QT syndrome; ITS = interrupted time series; ACA = aborted cardiac arrest; SCD = sudden cardiac death; ICD = implantable cardioverter defibrillator; VT = ventricular tachycardia; TdP = Torsades de Pointes; ND = not described; NS = not specified.

*others included acebutolol, bisoprolol, and pindolol.

### Effects of beta-blocker therapy on reduction of cardiac events

**Fig 2:**
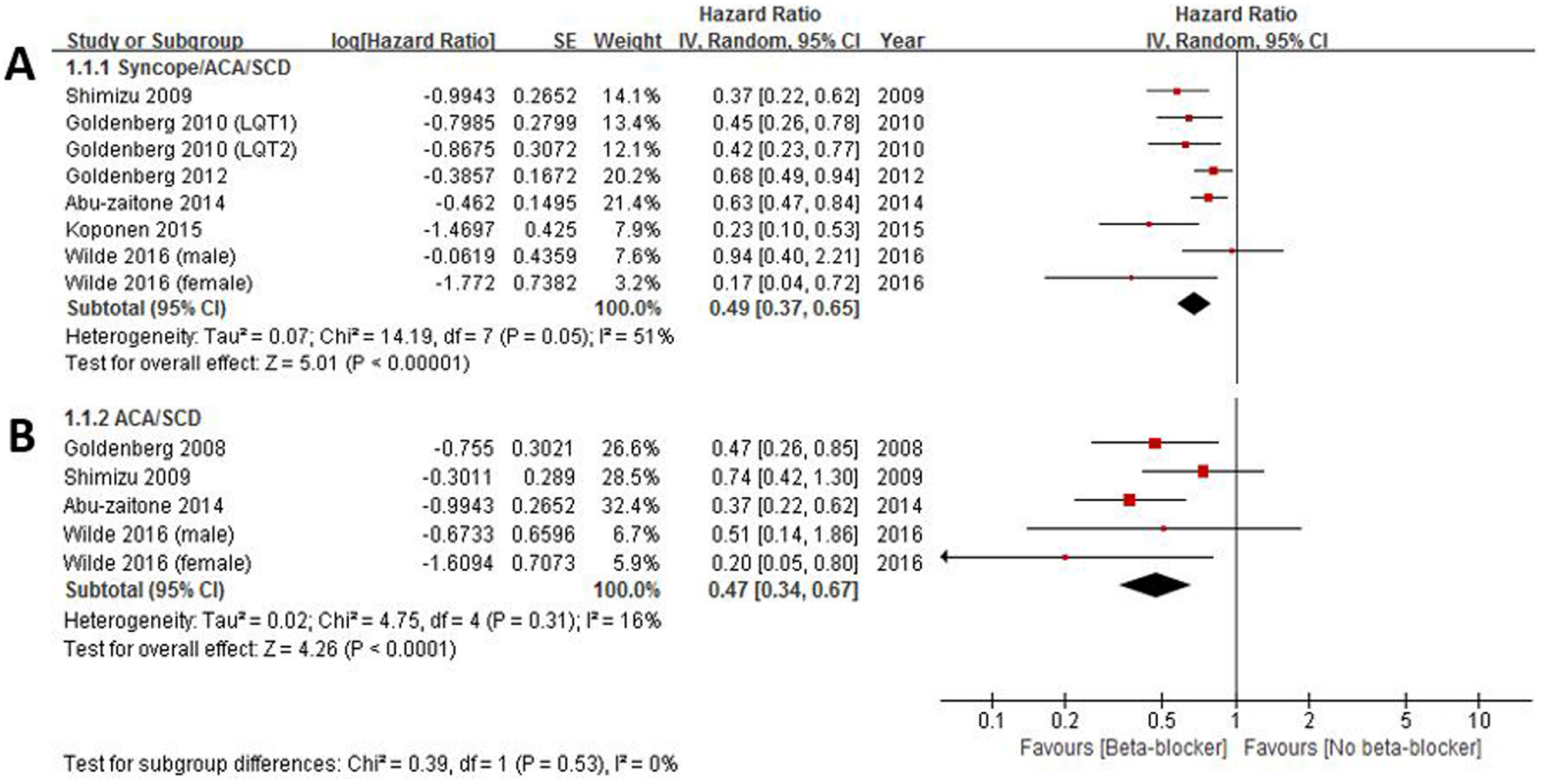
Effectiveness of beta-blockers on reduction of cardiac events. Cardiac events were defined as syncope, aborted cardiac arrest (ACA), or sudden cardiac death (SCD) (2A), and serious cardiac events were confined to ACA or SCD (2B).

In the cohort studies, 6 provided the multivariate-adjusted HRs for a composite of cardiac events including syncope, ACA, or SCD. The HRs of each study and all studies combined are shown in Fig 2. Overall, patients treated with beta-blocker compared with those with no beta-blocker showed a significant reduction in the risk of cardiac events (HR 0.49, 95% CI 0.37–0.65) (Fig 2A). There was no evidence of heterogeneity between the studies (*I*^2^ = 51%, p for heterogeneity 0.05) (Fig 2A). This effect of beta-blocker seemed to be stronger when a clinical outcome was confined to a composite of more serious cardiac events including ACA or SCA (HR 0.47, 95% CI 0.34–0.67, *I*^2^ = 16%, p for heterogeneity 0.31) (Fig 2B), but there was no statistical significance.

### Beta-blocker therapy according to long-QT genotype

**Fig 3:**
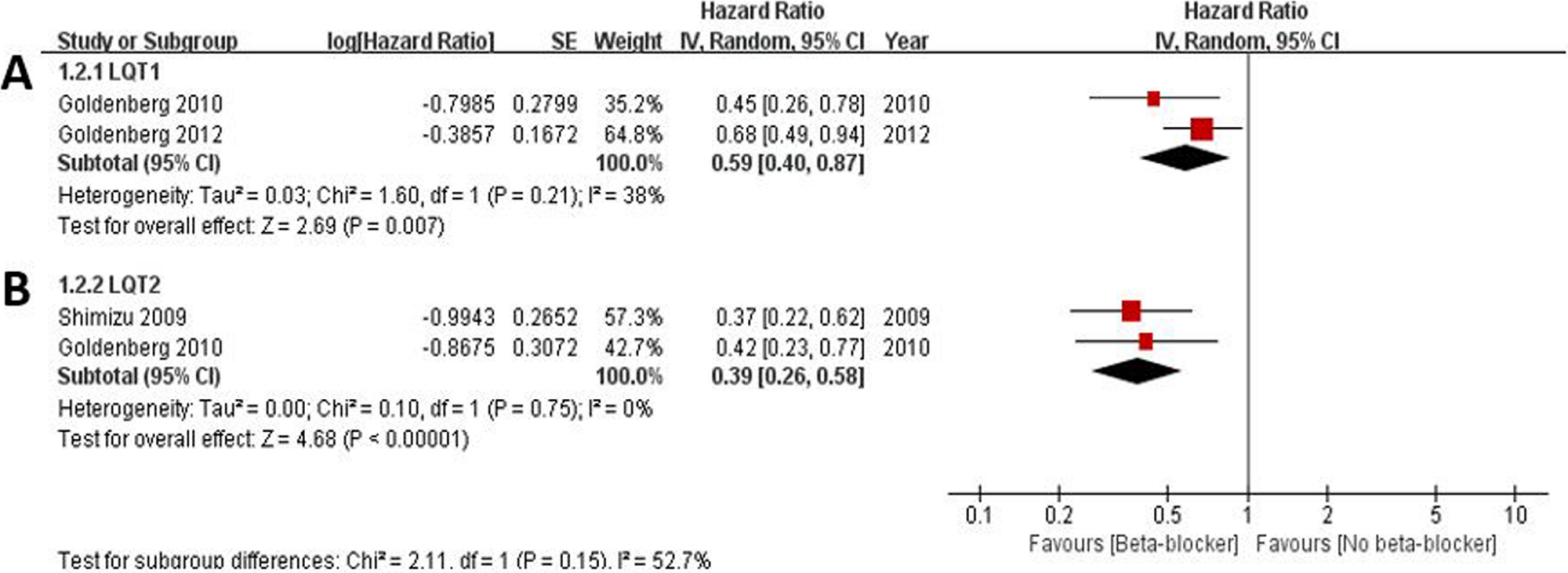
Effectiveness of beta-blockers on reduction of cardiac events according to long-QT syndrome (LQT) genotype. A. LQT type 1; B. LQT type 2.

In three out of 7 cohort studies, the effects of beta-blocker on cardiac events according to LQT subtypes were analyzed (Fig 3). The pooled HRs for cardiac events in beta-blocker treated groups compared to those without beta-blocker were 0.59 in LQT1 (95% CI 0.40–0.87, *I*^2^ = 38%, p for heterogeneity 0.21; Fig 3A) and 0.39 in LQT2 (95% CI 0.26–0.58, *I*^2^ = 0%, p for heterogeneity 0.75; Fig 3B), respectively.

### Effectiveness of different beta-blockers according to long-QT genotype

**Fig 4:**
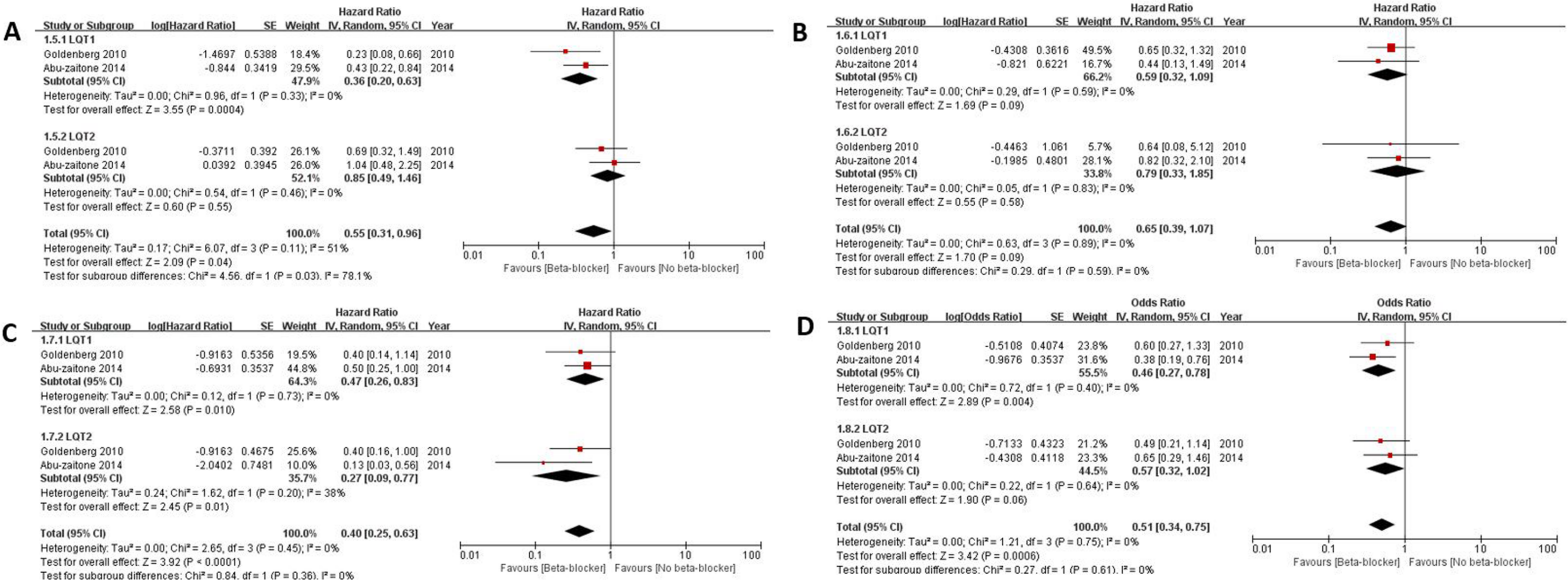
Comparison of effectiveness of beta-blockers on reduction of cardiac events between long-QT syndrome type 1 (LQT1) and 2 (LQT2). A. atenolol; B. metoprolol; C. nadolol; D. propranolol.

Different effects of various beta-blockers on the clinical outcomes were shown between LQT1 and LQT2. The prescribed beta-blockers included atenolol, nadolol, metoprolol, and propranolol. The pooled HR was calculated for each beta-blocker. Fig 4 shows forest plots for drug benefit according to LQT genotype. Atenolol significantly reduced cardiac events in LQT1 (HR 0.36, 95% CI 0.20–0.63, *I*^2^ = 0%, p for heterogeneity 0.33) compared to LQT2 (p for heterogeneity between two genotypes 0.03) (Fig 4A). Metoprolol showed a trend to decrease cardiac event only in LQT1, but there was no significant difference (Fig 4B). Nadolol showed significant risk reduction in both LQT1 (HR 0.47, 95% CI 0.26–0.83, *I*^2^ = 0%, p for heterogeneity 0.73) and LQT2 (HR 0.27, 95% CI 0.09–0.77, *I*^2^ = 38%, p for heterogeneity 0.20) (Fig 4C). Propranolol decreased the risk in only LQT1 (HR 0.46, 95% CI 0.27–0.78, *I*^2^ = 0%, p for heterogeneity 0.40) (Fig 4D).

The effects of each beta-blocker were also compared according to LQT genotype. In LQT1, atenolol, propranolol, and nadolol showed a similar magnitude of risk reduction of cardiac events (S2A Fig). In LQT2, nadolol was the only beta-blocker associated with a significant reduction in the risk of cardiac events (S2B Fig).

### Risk reduction of cardiac events before and after beta-blocker therapy: Interrupted time series study analysis

**Fig 5:**
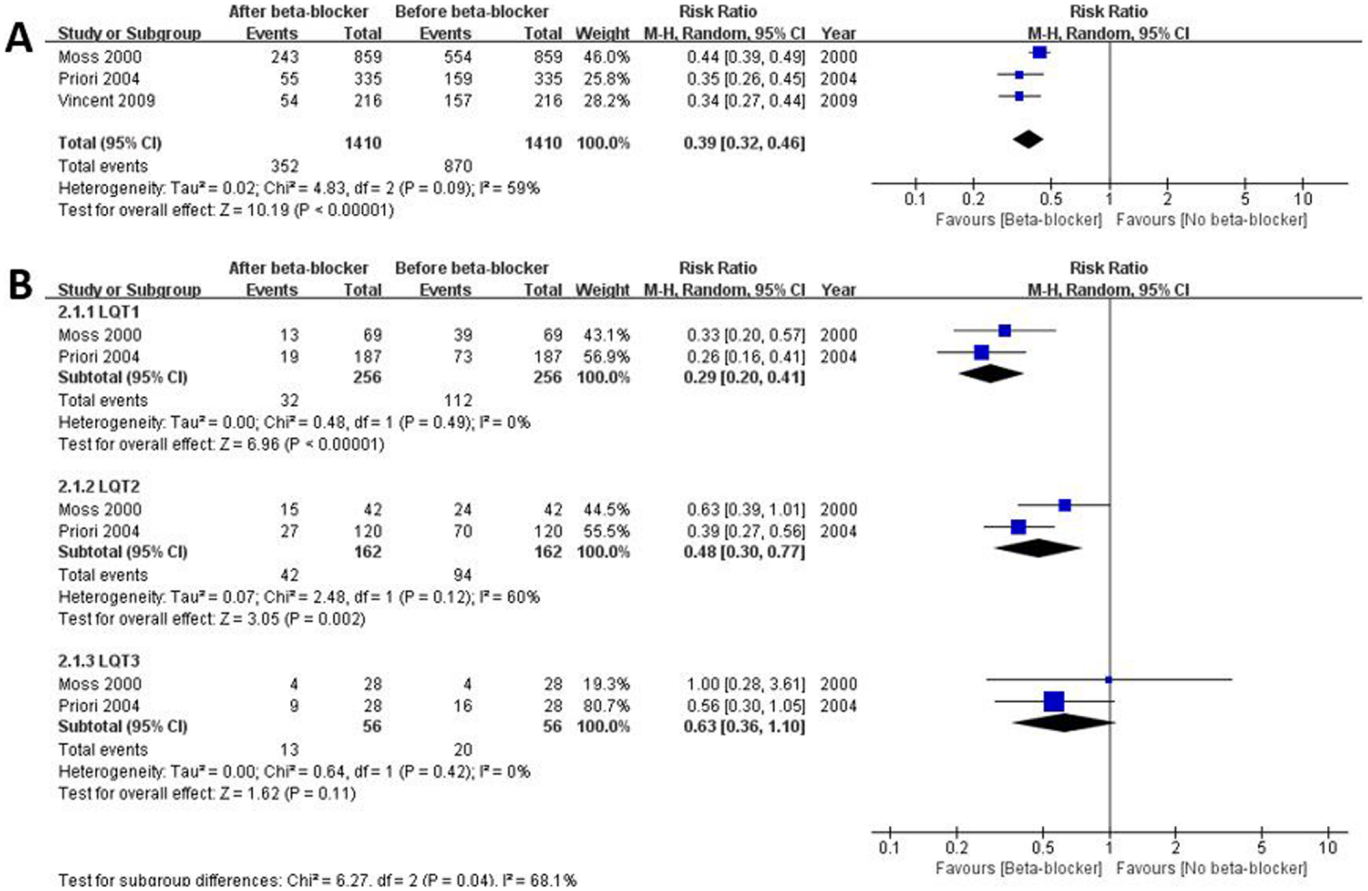
Risk reduction of cardiac events before and after beta-blocker therapy in the same population. A. All genotypes; B. comparison among genotypes. LQT1, long-QT syndrome type 1; LQT2, long-QT syndrome type 2; LQT3, long-QT syndrome type 3.

Three ITS studies were identified for the meta-analysis. Overall, among 1,410 participants, 870 events occurred before beta-blocker therapy, which was reduced significantly by 61% (352 events) after taking beta-blockers (RR 0.39, 95% CI 0.32–0.46, *I*^2^ = 59%, p for heterogeneity 0.09) (Fig 5A). In each LQT genotype, risk of cardiac events was reduced by 71% in LQT1 (RR 0.29, 95% CI 0.20–0.41, *I*^2^ = 0%, p for heterogeneity 0.49) and 52% in LQT2 (RR 0.48, 95% CI 0.30–0.77, *I*^2^ = 60%, p for heterogeneity 0.12) (Fig 5B). In LQT3, however, there was no significant difference in reduction of risk for cardiac events between before and after use of beta-blocker (Fig 5B). The effect of beta-blocker was better in patients with LQT1 than in those with LQT2 or LQT3 (S3 Fig).

## Discussion

### Main findings

To the best of our knowledge, this study is the first meta-analysis to review the evidence for effectiveness of various beta-blockers therapy according to congenital LQT genotype. The results of this meta-analysis including 7 Cohort and 3 ITS suggested that, overall, beta-blocker therapy was significantly associated with decreased risk for cardiac events (syncope, ACA, or SCD). However, this effect of beta-blocker was not the same for all kinds of beta-blockers or LQT genotypes. In addition, the effect of beta-blocker seemed to be stronger to reduce life-threatening, serious cardiac events (ACA or SCD) compared to all events including syncope.

### Effectiveness of therapy with beta-blockers depending on long-QT genotype

The three major genotypes of LQTS are LQT1, LQT2, and LQT3, which account for more than 80~90% of the mutations identified in congenital LQTS [2]. A predisposing genetic substrate is a potassium channel encoding *KCNQ1* and *KCNH2* or a sodium channel encoding *SCN5A* [2]. QT prolongation in LQTS might arise from prolongation of action potential duration due to either loss of function in slowly (*KCNQ1* mutation in LQT1) or rapidly (*KCNH2* mutation in LQT2) acting potassium outward current during phase 3 or to gain of function in late sodium inward current during the late plateau phase (*SCN5A* mutation in LQT3) [30]. Thus, identification of involved genes is important because there are some apparent data indicating a genotype-phenotype relationship, which affects clinical course and effectiveness of therapy among different genotypes [11, 31]. In LQT1, *KCNQ1* mutation causes reduction in *I*_Ks_ current, leading to prolonged duration of ventricular repolarization, which is aggravated by sympathetic stimuli [32]. Augmentation of sympathetic tone, such as exercise, is one of the important triggers of cardiac events and can be prevented by beta-blocking agents. Attenuation of transmural dispersion of repolarization and reduction of QT interval have been suggested as mechanisms of beta-blocking protection despite some controversies [33–35]. In LQT2, the effectiveness of beta-blockers has been thought to be less than that in LQT1 [8]. This difference might be caused by the presence of alpha_1A_ adrenoreceptor-mediated *I*_Kr_ reduction, leading to a relatively low incidence of cardiac events in younger patients through different patterns of predisposing triggers, such as emotional stress, auditory arousal, or rest, in LQT2 [2, 30]. However, this meta-analysis showed that the HR of beta-blockers on reduction of cardiac events had a trend toward being lower in LQT2 than in LQT1. Possible reasons for this observation are as follows: (1) In some portion of mutations, especially in non-pore-loop transmembraneous mutation, exercise-triggered events also occur in LQT2 [36]. (2) Two cohort studies in this meta-analysis showed 58% and 63% risk reduction in beta-blocker-treated patients with genetically confirmed LQT2 [28, 29]. The extents of risk reduction were greater compared with previous studies, which enrolled high-risk patients or participants in whom location, coding type, and topology were revealed but might have produced selection bias [8, 28, 29].

In LQT3, ITS studies showed that beta-blockers were not as effective as in LQT1 or LQT2. Analysis involving cohort studies could not be performed due to absence of more than two relevant cohort studies. However, the lack of sufficient data reflecting significant beta-blocker effects in LQT3 does not imply that beta-blockers should be prohibited in this genotype. While some previous small clinical or experimental studies suggested that beta-blockers were ineffective or even detrimental for this genetic subgroup [5, 8, 11, 37, 38], Calvillo et al. recently demonstrated that propranolol effectively prevented VT or VF in a validated LQT3 animal model [13]. Wilde et al. reported in their largest multicenter LQT3 cohort study that beta-blocker therapy significantly prevented cardiac events among treated females [14]. In addition, although bradycardia is considered the main concern in LQT3, one single *SCN5A* mutation can potentially cause several changes in sodium channels, and no apparent information regarding a relationship between this mutation and adrenergic mediation has been provided so far [39].

### Effects on reduction of cardiac events depending on beta-blocker

Even though beta-blockers have rightly constituted the first treatment of choice for cardiac events, earlier small studies suggested possible heterogeneous efficacies for different beta-blockers [10]. The main mechanism responsible for the reduction of cardiac events is considered their beta-adrenergic blockade effect, but pharmacological and pharmacokinetic properties are not the same for the various beta-blockers [15, 40, 41]. The present study showed that, in LQT1, atenolol, propranolol, and nadolol reduced cardiac events significantly compared with non-treated patients, whereas metoprolol did not. In LQT2, only nadolol showed a significant effect on reduction of cardiac events. The specific mechanism explaining these different effects was not determined, but some putative mechanisms can be suggested. First, metoprolol is a selective β1-adrenergic receptor antagonist and has minimal effects on peak or late sodium current [42]. Also, dosing might be an important factor since limited benefit was more frequent in high-risk patients who received metoprolol at a twice-daily dosing than at once-a-day dosing [41]. Second, nadolol does not have intrinsic sympathomimetic activity and is very long acting, which allows the most stable antiadrenergic activity. Also, it has peak sodium channel blocking effect like propranolol [12]. Third, unlike other beta-blockers, propranolol blocks *I*_kr_ at high concentration, which could result in less effectiveness in LQT2 [26, 40]. This is a plausible reason why propranolol performed badly in the New York registry study. Poor adherence to propranolol due to its rapid metabolization and non-selectivity might affect the outcome [18, 41]. On the other hand, both peak and late non-inactivating sodium current blocking effects of propranolol might be expected to show favorable effects to control cardiac events [43]. However, further studies are warranted to explain the exact mechanism of these findings.

### Effects of beta-blockers on serious cardiac events

The present study demonstrated risk reduction of cardiac events including or not including episodes of syncope in patients using beta-blockers. Even though the difference was not significant, the HR was slightly lower when the cardiac events were confined to ACA or SCD, suggesting that the extent of risk reduction was attenuated when syncope was included. Symptoms related to LQTS occurred for the first time by the age of 12 in 50% of patients and by the age of 40 in 90% [44]. Neurally-mediated syncope, such as vasovagal syncope, is also prevalent in young age, mostly before age 40 [45]. Some patients with LQTS have vasovagal syncope, which can be mistaken as LQTS-related syncope. Thus, it is difficult to distinguish between the causes of syncope. However, there is no evidence of benefit for use of beta-blockers in patients with vasovagal syncope aged younger than 40 years [45, 46]. Therefore, the effect of beta-blockers might be attenuated in patients with LQTS that present with syncope.

### Study limitations

This study has several limitations. First, the number of studies was relatively small, and most published reports were registry-based cohort studies. Because of the low prevalence of LQTS, it was very difficult to assess the efficacy of beta-blocker therapy through prospective randomized trials. Second, the effect of beta-blockers in LQTS can be influenced by various factors, such as age, gender, presence of prior events, QT interval, penetrance or combined intervention, adherence, adverse effects, and dosage regimen, such that inconsistencies might exist between studies [30]. However, this study showed acceptable heterogeneities in the measured values. Third, cross contamination among the registries is possible due to a limited number of studies. However, sensitivity analysis showed similar results. Fourth, although a recent study demonstrated acceptable effectiveness of bisoprolol than other beta-blockers [47], bisoprolol was not included in our data because of the small number of applicable patients.

## Conclusions

To the best of our knowledge, this study is the first meta-analysis assessing the effects of various beta-blockers in patients with LQTS according to genotype. Our study demonstrated the effectiveness of beta-blockers on reduction of cardiac events in LQTS, and that it was different depending on LQT genotype, suggesting that nadolol be considered first-line therapy, and that a genotype-guided strategy for the use of other beta-blockers can improve the clinical outcomes in patients with LQTS.

## Supporting information

S1 Checklist PRISMA checklist. (DOC)

S1 Fig Risk of bias assessment. Study quality was assessed using a Newcastle-Ottawa Quality Assessment Scale for cohort studies and 7 standard criteria suggested by Effective Practice and Organization of Care group for interrupted time series studies.
(TIF)

S2 Fig Comparison of effectiveness of various beta-blockers on reduction of cardiac events according to long-QT syndrome genotype. A. In LQT1, atenolol, propranolol, and nadolol showed similar degrees of risk reduction of cardiac events. B. In LQT2, only nadolol was associated with a significant reduction in the risk of cardiac events. LQT1, long-QT syndrome type 1; LQT2, long-QT syndrome type 2.
(EPS)

S3 Fig Comparison of effectiveness of beta-blockers between two long-QT syndrome genotypes from the interrupted time series studies. The effect of beta-blocker was better in patients with long-QT type 1 (LQT1) than in those with other types. A. LQT1 vs. Long-QT syndrome type 2 (LQT2); B. LQT1 vs. Long-QT syndrome type 3 (LQT3); C. LQT2 vs. LQT3.
(EPS)

S1 Table Database search strategy. Search terms used to select articles from the computerized databases MEDLINE, EMBASE, and the Cochrane Library (CENTRAL).
(DOCX)

## Abbreviations

ACA: aborted cardiac arrest
CI: confidence interval
Cohort: registry-based cohort studies
ECG: electrocardiogram
HR: hazard ratio
ITS: interrupted time series studies
LQT1: LQTS type 1
LQT2: LQTS type 2
LQT3: LQTS type 3
LQTS: long-QT syndrome
OR: odds ratio
PRISMA: Preferred Reporting Items for Systematic Reviews and Meta-Analyses
RR: relative risk
SCD: sudden cardiac death
