# Supplementary figures and images for: Effectiveness of beta-blockers depending on the genotype of congenital long-QT syndrome: A meta-analysis
Source: PLoS One. 2017 Oct 23;12(10):e0185680. doi: 10.1371/journal.pone.0185680 (PMC5653191; doi:10.1371/journal.pone.0185680)

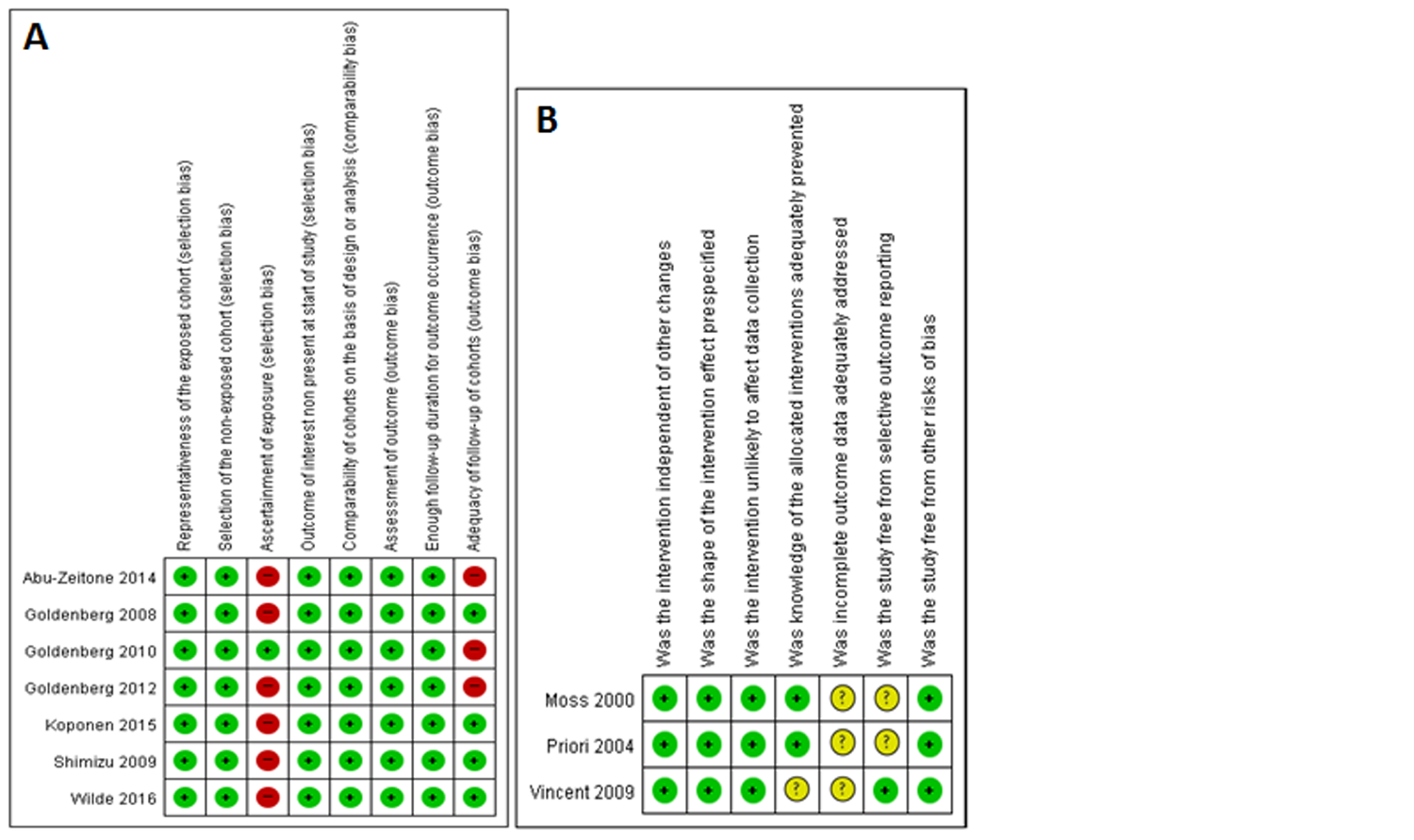

Supplement: S1 Fig — Study quality was assessed using a Newcastle-Ottawa Quality Assessment Scale for cohort studies and 7 standard criteria suggested by Effective Practice and Organization of Care group for interrupted time series studies. (TIF) [file pone.0185680.s002.tif]
