# Supplementary material for: Effectiveness of beta-blockers depending on the genotype of congenital long-QT syndrome: A meta-analysis
Source: PLoS One. 2017 Oct 23;12(10):e0185680. doi: 10.1371/journal.pone.0185680 (PMC5653191; doi:10.1371/journal.pone.0185680)
Supplement: S1 Table — Search terms used to select articles from the computerized databases MEDLINE, EMBASE, and the Cochrane Library (CENTRAL). (DOCX) [file pone.0185680.s005.docx]

**S1 Table. Database Searching strategy**

| **MEDLINE** |
| --- |
| 1. (((((((((((("Long QT Syndrome"[Mesh]) OR "Long Qt Syndrome 9" [Supplementary Concept]) OR "Long Qt Syndrome 12" [Supplementary Concept]) OR "Long Qt Syndrome 10" [Supplementary Concept]) OR "Long Qt Syndrome 11" [Supplementary Concept]) OR "Long Qt Syndrome 5" [Supplementary Concept]) OR "Long Qt Syndrome 2/5" [Supplementary Concept]) OR "Long Qt Syndrome 3/6" [Supplementary Concept]) OR "Long Qt Syndrome 6" [Supplementary Concept]) OR "Long Qt Syndrome 1/2" [Supplementary Concept]) OR "Long Qt Syndrome 2/3" [Supplementary Concept]) OR "Long Qt Syndrome 3" [Supplementary Concept]) OR "Long Qt Syndrome 2" [Supplementary Concept]) OR "Long Qt Syndrome 4" [Supplementary Concept]) OR "Long QT syndrome type 3" [Supplementary Concept] 6177 2. "Jervell Lange Nielsen Syndrome"[tiab] OR "Jervell-Lange Nielsen Syndrome"[tiab] OR "Andersen Syndrome"[tiab] OR "Andersen Tawil Syndrome"[tiab] OR "Andersen-Tawil Syndrome"[tiab] OR "Romano Ward Syndrome"[tiab] OR "Romano-Ward Syndrome"[tiab] OR "Ward-Romano Syndrome"[tiab] OR "Ward Romano Syndrome"[tiab] OR "long-QT syndrome"[tiab] OR "long QT syndrome"[tiab] OR "LQTS"[tiab] 4000 3. 1 OR 2 7487 4. Acebutolol[tiab] OR Alprenolol[tiab] OR amosulalol[tiab] OR arotinolol[tiab] OR Atenolol[tiab] OR befunolol[tiab] OR Betaxolol[tiab] OR bevantolol[tiab] OR Bisoprolol[tiab] OR bopindolol[tiab] OR bromoacetylalprenololmenthane[tiab] OR bucindolol[tiab] OR bufetolol[tiab] OR bufuralol[tiab] OR Bunolol[tiab] OR Bupranolol[tiab] OR butofilolol[tiab] OR Butoxamine[tiab] OR carazolol[tiab] OR Carteolol[tiab] OR carvedilol[tiab] OR Celiprolol[tiab] OR "CGP 12177"[tiab] OR "CGP 20712A"[tiab] OR cyanopindolol[tiab] OR Dihydroalprenolol[tiab] OR epanolol[tiab] OR esmolol[tiab] OR exaprolol[tiab] OR flestolol[tiab] OR "ICI 118551"[tiab] OR "ICI 89406"[tiab] OR indenolol[tiab] OR Iodocyanopindolol[tiab] OR "IPS 339"[tiab] OR Labetalol[tiab] OR landiolol[tiab] OR Levobunolol[tiab] OR medroxalol[tiab] OR mepindolol[tiab] OR Metipranolol[tiab] OR Metoprolol[tiab] OR "metoprolol succinate"[tiab] OR Nadolol[tiab] OR nebivolol[tiab] OR nipradilol[tiab] OR Oxprenolol[tiab] OR Penbutolol[tiab] OR Pindolol[tiab] OR Practolol[tiab] OR prizidilol[tiab] OR Propranolol[tiab] OR Sotalol[tiab] OR talinolol[tiab] OR tertatolol[tiab] OR tilisolol[tiab] OR Timolol[tiab] OR tobanum[tiab] 59047 5. ("Adrenergic beta-Antagonists"[Mesh]) OR "Adrenergic beta-Antagonists" [Pharmacological Action] 78711 6. "Adrenergic beta-Antagonists"[tiab] OR "Adrenergic beta Antagonists"[tiab] OR "beta-Adrenergic Receptor Blockaders"[tiab] OR "beta-Adrenergic Blockaders"[tiab] OR "beta Adrenergic Receptor Blockaders"[tiab] OR "beta-Adrenergic Blockers"[tiab] OR "beta Adrenergic Blockers"[tiab] OR "Adrenergic beta-Blockers"[tiab] OR "Adrenergic beta-Receptor Blockaders"[tiab] OR "Adrenergic beta Receptor Blockaders"[tiab] OR "beta-Adrenergic Blocking Agents"[tiab] OR "beta Adrenergic Blocking Agents"[tiab] OR "beta-blockers"[tiab] OR "β-blockers"[tiab] 19167 7. 4 OR 5 OR 6 102533 8. 3 AND 7 754 9. 8 NOT (animals[Mesh Term] NOT (humans[Mesh Term] AND animals[Mesh Term]) 673 |
| **EMBASE** |
| 1. 'long qt syndrome'/exp 8653 2. 'Jervell Lange Nielsen Syndrome':ab,ti OR 'Jervell-Lange Nielsen Syndrome':ab,ti OR 'Andersen Syndrome':ab,ti OR 'Andersen Tawil Syndrome':ab,ti OR 'Andersen-Tawil Syndrome':ab,ti OR 'Romano Ward Syndrome':ab,ti OR 'Romano-Ward Syndrome':ab,ti OR 'Ward-Romano Syndrome':ab,ti OR 'Ward Romano Syndrome':ab,ti OR 'long-QT syndrome':ab,ti OR 'long QT syndrome':ab,ti OR 'LQTS':ab,ti 5272 3. 1 OR 2 9482 4. Acebutolol:ab,ti OR Alprenolol:ab,ti OR amosulalol:ab,ti OR arotinolol:ab,ti OR Atenolol:ab,ti OR befunolol:ab,ti OR Betaxolol:ab,ti OR bevantolol:ab,ti OR Bisoprolol:ab,ti OR bopindolol:ab,ti OR bromoacetylalprenololmenthane:ab,ti OR bucindolol:ab,ti OR bufetolol:ab,ti OR bufuralol:ab,ti OR Bunolol:ab,ti OR Bupranolol:ab,ti OR butofilolol:ab,ti OR Butoxamine:ab,ti OR carazolol:ab,ti OR Carteolol:ab,ti OR carvedilol:ab,ti OR Celiprolol:ab,ti OR 'CGP 12177':ab,ti OR 'CGP 20712A':ab,ti OR cyanopindolol:ab,ti OR Dihydroalprenolol:ab,ti OR epanolol:ab,ti OR esmolol:ab,ti OR exaprolol:ab,ti OR flestolol:ab,ti OR 'ICI 118551':ab,ti OR 'ICI 89406':ab,ti OR indenolol:ab,ti OR Iodocyanopindolol:ab,ti OR 'IPS 339':ab,ti OR Labetalol:ab,ti OR landiolol:ab,ti OR Levobunolol:ab,ti OR medroxalol:ab,ti OR mepindolol:ab,ti OR Metipranolol:ab,ti OR Metoprolol:ab,ti OR 'metoprolol succinate':ab,ti OR Nadolol:ab,ti OR nebivolol:ab,ti OR nipradilol:ab,ti OR Oxprenolol:ab,ti OR Penbutolol:ab,ti OR Pindolol:ab,ti OR Practolol:ab,ti OR prizidilol:ab,ti OR Propranolol:ab,ti OR Sotalol:ab,ti OR talinolol:ab,ti OR tertatolol:ab,ti OR tilisolol:ab,ti OR Timolol:ab,ti OR tobanum:ab,ti 74422 5. 'beta adrenergic receptor blocking agent'/exp 245941 6. 'Adrenergic beta-Antagonists':ab,ti OR 'Adrenergic beta Antagonists':ab,ti OR 'beta-Adrenergic Receptor Blockaders':ab,ti OR 'beta-Adrenergic Blockaders':ab,ti OR 'beta Adrenergic Receptor Blockaders':ab,ti OR 'beta-Adrenergic Blockers':ab,ti OR 'beta Adrenergic Blockers':ab,ti OR 'Adrenergic beta-Blockers':ab,ti OR 'Adrenergic beta-Receptor Blockaders':ab,ti OR 'Adrenergic beta Receptor Blockaders':ab,ti OR 'beta-Adrenergic Blocking Agents':ab,ti OR 'beta Adrenergic Blocking Agents':ab,ti OR 'beta-blockers':ab,ti OR 'β-blockers':ab,ti 26307 7. 4 OR 5 OR 6 254805 8. 3 AND 7 2302 9. 8 NOT ('editorial'/it OR 'letter'/it OR 'note'/it OR 'short survey'/it) 2074 10. 9 NOT ('animal cell'/de OR 'animal experiment'/de OR 'animal model'/de OR 'animal tissue'/de OR 'in vitro study'/de OR 'nonhuman'/de) 1787 |
| **CENTRAL (Cochrane Library)** |
| 1. MeSH descriptor: [Long QT Syndrome] explode all trees 191 2. "Jervell Lange Nielsen Syndrome" or "Jervell-Lange Nielsen Syndrome" or "Andersen Syndrome" or "Andersen Tawil Syndrome" or "Andersen-Tawil Syndrome" or "Romano Ward Syndrome" or "Romano-Ward Syndrome" or "Ward-Romano Syndrome" or "Ward Romano Syndrome" or "long-QT syndrome" or "long QT syndrome" or "LQTS":ti,ab,kw 216 3. 1 OR 2 216 4. Acebutolol or Alprenolol or amosulalol or arotinolol or Atenolol or befunolol or Betaxolol or bevantolol or Bisoprolol or bopindolol or bromoacetylalprenololmenthane or bucindolol or bufetolol or bufuralol or Bunolol or Bupranolol or butofilolol or Butoxamine or carazolol or Carteolol or carvedilol or Celiprolol or "CGP 12177" or "CGP 20712A" or cyanopindolol or Dihydroalprenolol or epanolol or esmolol or exaprolol or flestolol or "ICI 118551" or "ICI 89406" or indenolol or Iodocyanopindolol or "IPS 339" or Labetalol or landiolol or Levobunolol or medroxalol or mepindolol or Metipranolol or Metoprolol or "metoprolol succinate" or Nadolol or nebivolol or nipradilol or Oxprenolol or Penbutolol or Pindolol or Practolol or prizidilol or Propranolol or Sotalol or talinolol or tertatolol or tilisolol or Timolol or tobanum:ti,ab,kw 14,558 5. MeSH descriptor: [Adrenergic beta-Antagonists] explode all trees 4,217 6. MeSH descriptor: [Adrenergic beta-Antagonists] explode all trees and with qualifier(s): [Pharmacology - PD] 1138 7. "Adrenergic beta-Antagonists" or "Adrenergic beta Antagonists" or "beta-Adrenergic Receptor Blockaders" or "beta-Adrenergic Blockaders" or "beta Adrenergic Receptor Blockaders" or "beta-Adrenergic Blockers" or "beta Adrenergic Blockers" or "Adrenergic beta-Blockers" or "Adrenergic beta-Receptor Blockaders" or "Adrenergic beta Receptor Blockaders" or "beta-Adrenergic Blocking Agents" or "beta Adrenergic Blocking Agents" or "beta-blockers" or "β-blockers":ti,ab,kw 7384 8. 4-7 / OR 16902 9. 3 AND 8 20 10. 8 / trials 19 |
| **Total number : MELINE 673 + EMBASE 1787 + CENTRAL 19 = 2,479**  **Duplication : 366**  **Final total number : 2,113** |
